# Extended metAFLP approach in studies of tissue culture induced variation (TCIV) in triticale

**DOI:** 10.1007/s11032-014-0079-2

**Published:** 2014-05-07

**Authors:** Joanna Machczyńska, Renata Orłowska, Janusz Zimny, Piotr Tomasz Bednarek

**Affiliations:** 1 Department of Plant Physiology and Biochemistry, Plant Breeding and Acclimatization Institute, National Research Institute, 05-870 Błonie, Radzików Poland; 2 Department of Plant Biotechnology and Cytogenetics, Plant Breeding and Acclimatization Institute, National Research Institute, 05-870 Błonie, Radzików Poland

**Keywords:** (Epi)genetics, Extended metAFLP, Tissue culture induced variation, Triticale

## Abstract

We present the development of the theoretical background of the metAFLP approach which allows for partition of complex variation into sequence changes, de novo methylation and demethylation of the regenerants derived via in vitro tissue culture methods in the case of triticale. It was demonstrated that, independent of whether andro- or embryogenesis was used for plant regeneration, the level of sequence changes identified between regenerants is about 10 %. Moreover, DNA demethylation prevails over de novo methylation of the regenerants compared to the donor plant. The metAFLP approach allows for the evaluation of numerous quantitative characteristics. For instance, one may quantify the number of sites unaffected by tissue culture approaches, global site DNA methylation etc. It is suggested that the approach could be useful for breeders in order to control plant material uniformity or for the evaluation of modified in vitro tissue culture approaches allowing for control of the (epi)mutation level. The extended metAFLP approach presented here delivers sufficient background for the evaluation of software that could facilitate analyses of the tissue culture induced variation.

**Electronic supplementary material:**

The online version of this article (doi:10.1007/s11032-014-0079-2) contains supplementary material, which is available to authorized users.

## Introduction

(Epi)genetic changes can be introduced into the genome during in vitro tissue culture plant regeneration. Alterations induced at the level of regenerants are usually called tissue culture induced variation (TCIV) (Bednarek et al. 2007). If stably transmitted to the next generations (Neelakandan and Wang 2012) they would be recognized as somaclonal variation (SV) (Bednarek et al. 2007). In some instances, off-type regenerants and/or their progeny can be observed (Kaeppler and Phillips 1993). Even if morphological, cytological or physiological changes, for example, are not detected among regenerants, some variation may be exhibited spontaneously during successive generative cycles (Puente et al. 2008). Moreover, changes induced among regenerants (Bednarek et al. 2007) may segregate in an unpredictable manner (Liu and Wendel 2002) and are usually related to epimutations that could have occurred due to DNA methylation pattern alterations (Matthes et al. 2001; Peredo et al. 2009) or activation of retrotransposons (Liu et al. 2004; Ngezahayo et al. 2009). When homozygous plant material is required, e.g. for breeding purposes, it is preferential to control the level of (epi)mutations arising from regenerants. Thus, approaches capable of their identification and quantification are needed.

Quantitative characteristics of DNA methylation patterns can be evaluated using high-performance liquid chromatography (HPLC) (Parra et al. 2001). While useful for general purposes, this cannot detect subtle effects such as DNA demethylation or de novo methylation. Moreover, HPLC is not adequate for the quantification of changes in DNA sequence and does not deliver information on their chromosomal location. Such disadvantages can be avoided using marker-based approaches. To date, TCIV has been studied using the *Hpa*II and *Msp*I isoschizomers (Matthes et al. 2001; Dann and Wilson 2011). Although highly useful (Miguel and Marum 2011), this approach is limited to specific restriction sites. Moreover, it can evaluate only some types of methylation changes (Schulz et al. 2013) and may cause problems when identifying sequence variation in a single experiment (Pérez-Figueroa 2013). Additionally, it does not allow the quantification of TCIV. The gap can be partially filled by the metAFLP approach (Bednarek et al. 2007), which has proved to be useful for the quantification of (epi)genetic changes induced among regenerants during in vitro tissue culture manipulation, if the appropriate plant material is used. The interpretation of the molecular profiles amplified in *Acc65*I/*Mse*I and *Kpn*I/*Mse*I AFLP platforms for the donor plant and its regenerants allowed classification of the molecular patterns into sequence changes, de novo methylation and demethylation in a single experiment.

At a very preliminary stage of development, the metAFLP approach was applied to study TCIV in barley (Bednarek et al. 2007), *Gentiana pannonica* (Fiuk et al. 2010) and *Phyllostachys praecox* (Lu et al. 2012). MetAFLP platforms proved to be a valuable tool for analyzing changes induced by cryopreservation in *Gentiana cruciata* tissue (Mikuła et al. 2011), or for evaluating the genetic distinctiveness of metallicolous and non-metallicolous populations of *Armeria maritime* (Abratowska et al. 2012). Population genetic analysis of *Poa annua* (Chwedorzewska and Bednarek 2012) demonstrated that the approach could be used to extract information on epigenetic variation even in the case of population studies. Evidently, progress in the development of the approach to evaluate additional characteristics is possible, but the theoretical background of the model needs further attention.

The primary aim of the study was further development of the theoretical background of the metAFLP approach that could support additional characteristics of TCIV and sites unaffected by any changes, in order to deliver an extended molecular marker-based tool for studies of changes induced during in vitro tissue culture manipulations. The usability of the extended method is demonstrated based on triticale plant materials designed for the given study and not previously analyzed.

## Materials and methods

### Plant material

Doubled haploid donor plants derived from isolated microspores of winter triticale cv. Bogo (Oleszczuk et al. 2004) served as a source of tissues to obtain regenerants via androgenesis and somatic embryogenesis (Online Resource 1). In the case of androgenesis, both anther and shed-microspore cultures were used to evaluate regenerants, while in somatic embryogenesis immature zygotic embryo cultures were involved.

### Androgenesis and somatic embryogenesis

The tillers of doubled haploid donor plants with the microspore at the mid to late uninucleate stage were cut and kept in a cool room for 4 weeks. Anthers from one half of the sterilized spikes were then excised and placed on 190-2 regeneration medium (Zhuang and Xu 1983) together with 90 g/L maltose, 400 mg/L glutamine, 2 mg/L 2,4-D and 0.5 mg/L kinetin solidified with Phytagel, whereas anthers from the second half of the same spikes were placed on the same but liquid regeneration medium. To induce androgenesis, anthers on both solid and liquid medium were kept in the dark for 6–8 weeks at 26 °C. Afterwards, appearing embryo-like structures and calli from solid and liquid medium were transferred onto 190-2 regeneration medium with 0.5 mg/L NAA and 0.5 mg/L kinetin. Plantlets were subsequently transferred to rooting medium (190-2 with 2 mg/L IAA). Small plants derived from anther cultures on solid regeneration medium as well as plants derived from shed-microspore cultures on liquid medium were kept at 4 °C for 6 weeks, following their acclimatization in soil.

To induce somatic embryogenesis, sterilized zygotic embryos at the coleoptile stage were transferred onto MS medium with 30 µM dicamba (Murashige and Skoog 1962). After ca. 4 weeks of culture, somatic structures were transferred on the MS medium with 0.9 µM 2,4-D. Germinating somatic embryos were then placed on the 190-2 regeneration medium, following their transfer to root formation medium (both media were the same as in androgenesis). In the next step, small plantlets were vernalized in a cool room for 6 weeks, following their adaptation to soil in a greenhouse.

### DNA isolation

Fresh leaves of donor plants and their doubled haploid regenerants at the same developmental stage were cut and used for genomic DNA isolation according to the procedure described in DNeasy Plant Mini Kit (Qiagen). The samples were characterized spectrophotometrically following verification of their integrity and purity on agarose gels.

### MetAFLP approach

The detailed procedure of the metAFLP experiment was described in our previous study (Bednarek et al. 2007). Briefly, equal aliquots of the same DNA samples were digested with *Acc65*I/*Mse*I and *Kpn*I/*Mse*I endonucleases, respectively. Adaptors (Online Resource 2) were added to the reaction mixtures following their ligation to the digested DNA fragments. The reaction mixtures were diluted with water (1:10) and used for pre-selective amplification with pre-selective primers. After pre-selective amplification the reaction mixtures were again diluted with water (1:20) and used for selective amplification in the presence of ^32^P-labelled selective primer complementary to the adaptor sequence of the *Acc65*I–*Kpn*I sequence. Finally, the reaction mixture was fractionated on 7 % PAGE (polyacrylamide gel) and DNA markers were visualized on X-ray films.

### MetAFLP background and formulae used to calculate different parameters

The metAFLP approach is based on two isoschizomers: *Acc65*I and *Kpn*I. They recognize the same restriction sequence but differ in their sensitivity towards DNA methylation of their recognition sequences; the former is sensitive to cytosine methylation of the restriction site and its adjacent sequences whereas the latter is not (Online Resource 3). The juxtaposed AFLP profiles generated in the *Acc65*I/*Mse*I and *Kpn*I/*Mse*I platforms are used for marker counting. If the restriction site is affected by methylation, the two platforms will amplify different DNA fragments from the same DNA matrix. The molecular profiles amplified on donor (D) and regenerant (R) DNAs in both AFLP platforms can be encoded in the form of a 4-digit binary code. The first two digits reflect the presence (1) or absence (0) of a marker in the *Acc65*I/*Mse*I platform for D and R while the second two encode the markers for D and R in the *Kpn*I/*Mse*I one. There are 16 permutations of the code (all possible combinations of 0 and 1 in four positions). Each 4-digit code reflects a specific genetic background of sequence, demethylation and de novo methylation events as well as, for example, sites unaffected by any change between donor and regenerant. Codes reflecting the same event type combined are used to deliver quantitative characteristics of the metAFLP approach. The genetic background allowing interpretation of codes and their classification of different event types has been discussed previously (Bednarek et al. 2007). The details of the formulae for the calculation of different metAFLP events are presented in Table 1.

**Table 1:** Formulae used in metAFLP approach

Abbreviation	Formula	Description
SE	∑(0001) + ∑(0010) + ∑(0101) + ∑(0110) + ∑(1001) + ∑(1010) + ∑(1110) + ∑(1101)	Sequence events
DME	∑(0111) + ∑(0110)	Demethylation events
DNME	∑(1011) + ∑(1001)	De novo methylation events
CE	∑(0100) + ∑(1000)	Complex events
SNMS	∑(1100) + ∑(1111) + ∑(1101)	Sites with non-methylated status in D and R (‘inherited’ non-methylated status)
SMS	∑(0011) + (1100)	Sites with methylated status in D and R (‘inherited’ methylated status)
TNTCIE	SE +DME + DNME + CE	Total number of tissue culture induced events

All the event types can be converted into variations after dividing them by the denominator 1, which is the sum of TNTCIEs, SNMSs and SMSs, as well as the ‘0000’ code (Table 2) expressed in percentages. Although the ‘0000’ code is meaningless, it can be present in the case of, e.g. a D–R_*i*_ pair, but is not necessary in D–R_*n*+1_ (where the subscript *i* denotes a successive regenerant). Its incorporation into the formula is conditioned by the simplicity of calculations if many regenerants (or different donors and their regenerants) are being analyzed simultaneously. Moreover, if it is included, the sum of all TNTCIEs as well as other metAFLP characteristics, such as the number of sites with methylated/nonmethylated status shared between D and R (SMSs/SNMSs), plus ‘0000’ cases is equal to 100 %.

**Table 2:** Formulae used to calculate basic metAFLP characteristics

Abbreviation	Formula	Description
DENOMINATOR1	SE + DME + DNME + CE + SMSs + SNMSs + 0000	Used to calculate percentages of SV, DMV, DNMV, CV, SNMS, SMS and TTCIV
SV	100 × SE/DENOMINATOR1	Sequence variation
DMV	100 × DME/DENOMINATOR1	Demethylation variation
DNMV	100 × DNME/DENOMINATOR1	De novo methylation variation
CV	100 × CE/DENOMINATOR1	Complex variation
TTCIV	100 × TTCIE/DENOMINATOR1	Total tissue culture induced variation
SNMS	100 × SNMSs/DENOMINATOR1	‘Inherited’ non-methylation
SMS	100 × SMSs/DENOMINATOR1	‘Inherited’ methylation

One of the unsolved problems in our previous study (Bednarek et al. 2007) was the complex variation (CV), which was made up from four events encoded as ‘0100’, ‘1001’, ‘0110’ and ‘1000’. Such codes could be explained by different events that might have happened independently or simultaneously. Previously, we have assumed that such cases are rare and may not affect calculations significantly. While this is in general true, as demonstrated in the case of barley, it became evident that some variation types might have had quite considerable values. Thus, it may be problematic to ensure that the differences between them or between analogue data sets are significant. Analyses of the complex events demonstrated that two of them (‘1001’ and ‘0110’) could happen only if two strictly defined events took place simultaneously. For example, code ‘1001’ is explained by de novo methylation and sequence change (Online Resource 4) while ‘0110’ by demethylation and sequence event (Online Resource 5). Thus, the events encoded by the abovementioned codes could be reclassified to de novo methylation, demethylation and sequence change, leading to modifications of the formulae for SE, DME, DNME and CE in the extended metAFLP approach (Table 1) in comparison to those presented earlier (Bednarek et al. 2007).

Further partition of the complex events was possible at the level of variation. Assuming that sequence variation (SV), demethylation (DMV) and de novo demethylation (DNMV) are proportionally represented in complex variation (represented by different event types), one may calculate correction coefficients that allow subdivision of CV into SV, DMV and DNMV (correction coefficients in Table 3). If CCSV, CCDMV and CCDNMV are added to SV, DMV and DNMV, the corrected values of sequence variation (CSV), demethylation (CDMV) and de novo methylation (CDNMV) can be calculated (Table 3). Such reasoning resulted in complete partition of the CV.

**Table 3:** Formulae for correction coefficients and corrected variation types

Abbreviation	Formula	Description
CCSV	CV × SV/(SV + DMV + DNMV)	Correction coefficients SV
CCDMV	CV × DMV/(SV + DMV + DNMV)	Correction coefficients DMV
CCDNMV	CV × DNMV/(SV + DMV + DNMV)	Correction coefficients DNMV
CSV	SV + CCSV	Corrected SV
CDMV	DMV + CCDMV	Corrected DMV
CDNMV	DNMV + CCDNMV	Corrected DNMV

Moreover, the metAFLP approach allows evaluation of many useful characteristics describing regenerants derived from tissue cultures (Table 4). For instance, the total number of non-methylated (TNNMSs) and methylated (TNMSs) restriction sites can deliver information on the number of restriction sites that were either non-methylated or methylated in regenerants. As TNNMSs and TNMSs combined reflect all restriction sites analyzed, global non-methylation (GNM) and global DNA methylation (GM) can be expressed as percentages. It is also possible to quantify the number of restriction sites unaffected by total tissue culture induced variation (SUTCIV). Knowing TTCIV and corrected sequence variation (CSV), the information on the amount of variation related to changes in DNA methylation can be evaluated (SAM).

**Table 4:** Characteristics describing tissue culture derived regenerants based on analysis of metAFLP profiles

Abbreviation	Formula	Description
TNNMSs	DME + SNMSs	Total number of non-methylated sites
TNMSs	DNME + SMSs	Total number of methylated sites
DENOMINATOR2	TNMSs + TNNMSs	Used to calculate percentages of GM and GNM
GM	100 × TNMSs/DENOMINATOR2	Genome methylation
GNM	100 × TNNMSs/DENOMINATOR2	Genome non-methylation
SUTCIV	100 − TTCIV	Sites unaffected by TTCIV
SAM	TTCIV − CSV	Sites affected by methylation

### Statistics

ANOVA was used to test differences between variation types. Analysis was carried out using R software.

## Results

A homozygous plant was used as the source of tissues for plant regeneration via in vitro tissue culture approaches. For the present studies we used 25, 30 and 26 plants derived from anther cultures, shed-microspore cultures and immature zygotic embryos, respectively. No morphological variation or off-type regenerants were observed among spontaneously doubled plants as well as among plants derived via embryogenesis.

As many as 14 selective primer combinations were used (Online Resource 6) to analyze the donor plant and its regenerant DNAs with the metAFLP approach. There were 584 bands shared between *Acc65*I/*Mse*I and *Kpn*I/*Mse*I AFLP platforms, with an average number per selective primer combination of 41.7. Two hundred and twenty-two DNA fragments were monomorphic in both cases while 274 and 306 were monomorphic in one of the platforms.

Conversion of the metAFLP 4-digit codes of the regenerants derived from each tissue culture approach, independently and for all of them taken together (Online Resource 7), demonstrated that the events classified as ‘0000’ and ‘1111’ were the most while ‘1001’ and ‘0110’ were the least frequent.

Quantitative characteristics of the metAFLP approach for the regenerants derived via the three approaches are listed in Table 5. Sequence variation, demethylation and de novo methylation uncorrected for complex variation amounted to as much as 10.1, 3.97 and 2.82 % of variation, respectively. Introduction of the correction coefficients resulted in an increase of the quantitative characteristics for sequence variation and increased the difference between demethylation and de novo methylation. Independently of the regeneration method, the level of corrected sequence variation was relatively high and ranged around 11 %. Corrected demethylation seemed to be highest in the case of the regenerants derived via embryogenesis, and lowest for the shed-microspore cultures, while corrected de novo methylation of the restriction sites was observed to be highest for the shed-microspore-derived regenerants and lowest for those related to anther cultures. The average levels of corrected demethylation and de novo methylation were 4.4 and 3.1 %, respectively. Total tissue culture induced variation was highest for the regenerants derived via embryogenesis and lowest in the case of the anther cultures. Nevertheless, the average level of TTCIV was high and amounted to 19 %. Quantification of the restriction sites affected by methylation, reflecting sites that preserved methylation status between D and R, sites that underwent demethylation and those that underwent de novo methylation demonstrated that these characteristics ranged around 7.6 %. Interestingly, the level of methylated restriction sites that preserved their methylation status (SMS) among regenerants was relatively low, as only 3.7 % of sites exhibited no change in methylation pattern compared to the donor plant. Moreover, androgenesis-derived regenerants seemed to preserve a higher number of methylated sites than the regenerants obtained from immature embryos. On the other hand, quantification of the non-methylated restriction sites that did not change their status in regenerants (SNMS) was comparable between all approaches and was about 70 %. Quantification of genome methylation (GM) based on the metAFLP profiles and reflecting the percentages of methylated restriction sites identified among regenerants demonstrated that the triticale genome of the regenerants may be methylated in 8 %, with the highest value of this parameter in the case of shed-microspore-derived regenerants.

**Table 5:** The percentage of changes induced during in vitro culture plant regeneration in triticale cv

metAFLP characteristics	Quantitative characteristics of regenerants within regeneration method			Quantitative characteristics of all regenerants taken together
	RA	RM	RE	∑
Sequence variation	9.79 ± 0.84	10.2 ± 1.44	10.3 ± 0.66	10.1 ± 1.07
Demethylation	3.99 ± 0.48	3.59 ± 0.5	4.38 ± 0.3	3.97 ± 0.54
De novo methylation	2.52 ± 1.05	3.15 ± 0.89	2.73 ± 0.72	2.82 ± 0.92
Complex variation	2.05 ± 0.46	2.3 ± 0.63	1.98 ± 0.46	2.12 ± 0.54
Corrected sequence variation	11.02 ± 0.93	11.62 ± 1.62	11.49 ± 0.62	11.4 ± 1.18
Corrected demethylation	4.5 ± 0.54	4.07 ± 0.69	4.88 ± 0.32	4.46 ± 0.60
Corrected de novo methylation	2.84 ± 1.14	3.58 ± 0.99	3.04 ± 0.79	3.18 ± 1.02
Total tissue culture induced variation	18.36 ± 1.43	19.28 ± 2.14	19.41 ± 0.91	19.04 ± 1.65
Sites affected by methylation	7.34 ± 1.07	7.66 ± 1.18	7.92 ± 0.85	7.64 ± 1.06
Sites unaffected by TTCIV	81.64 ± 1.43	80.72 ± 2.14	80.59 ± 0.91	80.96 ± 1.65
‘Inherited’ methylation	3.94 ± 0.68	4.29 ± 0.67	2.97 ± 0.32	3.76 ± 0.8
‘Inherited’ non-methylation	70.8 ± 1.05	69.71 ± 1.29	70.56 ± 0.85	70.32 ± 1.17
Genome methylation	7.95 ± 1.84	9.21 ± 1.34	7.07 ± 1.11	8.14 ± 1.74
Genome non-methylation	92.05 ± 1.84	90.79 ± 1.34	92.93 ± 1.11	91.86 ± 1.74

Bogo. RA, RM, RE: regenerants derived from anther cultures, shed-microspore cultures and immature zygotic embryo cultures, respectively (± corresponds to standard error)

Analysis of variance (Online Resource 8) demonstrated that the models explaining differences between uncorrected and corrected for CV variations were significant. However, *F* statistics for the latter, independently of whether plant regeneration method was considered or not, were higher. Based on Tukey’s test, the differences between SV, DMV and DNMV as well as between CSV, CDMV and CDNMV were significant (Online Resource 9) and distinguished all variation types except DMV(CDMV) and DNMV(CDNMV) in the case of shed-microspore-derived regenerants.

## Discussion

The genetic model of the metAFLP approach is based on the fact that a highly homozygous donor plant is used as the source of tissue cultures for in vitro plant regeneration. It is also assumed that any changes evaluated between regenerants are related to tissue culture induced variation. This assumption is valid, since if tissue cultures are not responsible for the changes then the regenerants derived from homozygous tissue should be identical with its donor plant. Moreover, in the given case, the putative difference between regenerants should not be ascribed to recombination events since they should not result in any variation in the case of homozygous materials. Thus, any variation between the donor plant and its regenerant evaluated with the metAFLP approach is induced by in vitro tissue culture regeneration and can be quantified based on molecular profiles.

The metAFLP approach in its preliminary form allowed for the quantification of the TCIV types related to sequence variation, demethylation, de novo methylation and complex variation. Analysis of their genetic background demonstrated that complex variation resulted from different types of changes that could have originated independently or simultaneously, which is why its partition into strictly defined variation types was complicated. Moreover, the level of complex variation was relatively low, as indicated in studies on barley (Bednarek et al. 2007). It was assumed that even if not used for quantification purposes, it should not significantly affect the characteristics of other variation types. On the other hand, partition of that variation may improve the reliability of statistical analyses in the case when some variation types have comparable values, or when the same or similar results for different data sets are generated or subtle effects of low frequency are being investigated. Evidently, complex variation could be eliminated from the analyses since two types of events classified as CE in our previous study (Bednarek et al. 2007) are strictly defined by two events that need to take place simultaneously. Thus, they could be reclassified into sequence change, demethylation and de novo methylation. Further elimination of the complex variation encompassing only two events (‘1000’ and ‘0100’) could be performed assuming that each variation type in CV is represented proportionally to its uncorrected type. The prerequisite for such an assumption was the fact that the probability of different variations encompassing CV should be the same as with SV, DMV and DNMV.

In the present study, the quantitative characteristics of uncorrected demethylation and de novo methylation evaluated for triticale were relatively close to each other. However, introduction of the correction coefficients allowed for their better differentiation (as indicated by ANOVA *F* statistics), while CDNMV and CDMV in RM remained in the same group based on Tukey’s grouping. It was also demonstrated that the level of sequence variation uncorrected for CV was underestimated. Comparison of the preliminary results for triticale and barley (Bednarek et al. 2007) demonstrates that the two genomes behave differently under in vitro tissue culture plant regeneration. One of the main differences is related to sequence changes, which in triticale are higher than in barley. Moreover, in triticale the level of demethylation is higher than in barley. Although the result given is just an illustration of the extended version of the metAFLP approach (the experimental study is under preparation), the observed differences may reflect problems with genome stability recognized in triticale (Appels et al. 1982). Many authors have demonstrated that triticale is affected by numerous chromosomal rearrangements (Tang et al. 2008; Oleszczuk et al. 2011) mostly influencing the rye genome (Charmet et al. 1986). It was also suggested that rye chromosomes in triticale become more homozygous than in *Secale cereale* L., possibly due to the elimination of retrotransposons during genome polyploidization (Bento et al. 2008). Possibly, stressful conditions, such as in vitro tissue culture plant regeneration, may activate mobile elements resulting in increased sequence variation. Alternatively, tissue cultures may result in abiotic stresses influencing the replication system, stimulating DNA polymerase slippage-related mutations (Alhani and Wilkinson 1998). Preliminary experimental data demonstrates that in triticale, independently of the tissue culture approach used for plant regeneration, genome demethylation of the restriction sites prevails over de novo DNA methylation. Such a behavior seems to be correlated with the known action of abiotic stresses that usually lead to genome demethylation (Choi and Sano 2007; Fan et al. 2013). Possibly, reprogramming of the tissues requires increased genome demethylation in order to activate previously methylated genes expressed during plant regeneration. Interestingly, the highest value of demethylation was evaluated for the embryo-derived regenerants. One may speculate that the presence of callus phase, which is usually thought to be the source of variation, was induced in in vitro tissue cultures (Piccioni et al. 1997). The present data are in disagreement with the results in barley (Bednarek et al. 2007), where genome de novo methylation prevailed. The current discrepancy is not clear, but one may speculate that complex triticale genome and genome conflicts may play a crucial role here.

Moreover, metAFLP may deliver information on the number of restriction sites that preserve their methylation or non-methylation status between the donor plant and its regenerant. Based on the current data, the level of methylated sites that did not change their methylation status between donor plant and its regenerant amounted to 3.7 % while global genome methylation (GM) accounts for about 8 %. The observed low level of methylated sites that did not change their methylation status as well as the level of CDNMV and CDMV demonstrated that in vitro tissue cultures induce numerous epimutations in triticale. The presence of those changes may explain the spontaneous erasure of off-type plants described by breeders (personal communication) and presented in the literature (Jaligot et al. 2004).

It would be interesting to verify how much the GM value reflects genome global methylation based on the HPLC approach. Such a comparison would allow estimation of to what extent the metAFLP GM value reflects genome-wide DNA methylation. If available, such data would demonstrate whether metAFLP results related to DNA methylation are representative of the whole genome. It is usually assumed that restriction sites are more or less randomly distributed along chromosomes. However, at the moment there is no information on the distribution of the metAFLP markers on either rye or wheat chromosomes. Nevertheless, mapping data in rye performed with an *EcoR*I/*Mse*I AFLP platform (Bednarek et al. 2002) showed that the AFLPs formed clusters of tightly linked markers with numerous gaps. Most probably, the same is true in the case of metAFLPs. Thus, one may expect the quantitative metAFLP characteristics related to methylation to be underestimated.

The advantage of the extended metAFLP approach is that it allows for better analysis of subtle effects (demethylation vs. de novo methylation) not evaluated in such a way until now. For example, HPLC can only deliver information on general changes in DNA methylation (Fraga et al. 2002) and cannot quantify both effects separately, while in the MSAP approach no unanimity exists on how to interpret and score the banding patterns that refer to methylation events (Schulz et al. 2013). Moreover, the metAFLP approach is capable of quantifying numerous additional characteristics describing either dynamic (epi)genetic changes (SV, DNMV, DMV) as well as static parameters (SMS, SNMS) that are not evaluated by any other molecular marker-based system.

## Conclusions

The extended metAFLP approach can be used for studies of TCIV, as demonstrated in triticale, giving increased resolution between variation types after partition of the complex variation. In vitro tissue cultures were responsible for the induction of a high level of sequence variation as well as for the changes in genomic DNA methylation patterns evaluated among regenerants. Moreover, the method reveals numerous quantitative characteristics (e.g. SMSs, SNMSs, GM, GNM etc.) describing plants derived via tissue cultures. These characteristics may be valuable for breeders as they could be used for the characterization of the uniformity of the regenerants. They could be also used for the evaluation of modified in vitro tissue culture approaches that may limit (epi)mutations or studies of abiotic and biotic stresses if appropriate plant materials and control conditions are applied. The present model could be suitable for implementation in a software package that would facilitate evaluation of quantitative characteristics.

## Electronic supplementary material

Below is the link to the electronic supplementary material.

Supplementary material 1 (PDF 227 kb)

Supplementary material 2 (PDF 213 kb)

Supplementary material 3 (PDF 187 kb)

Supplementary material 4 (PDF 204 kb)

Supplementary material 5 (PDF 131 kb)

Supplementary material 6 (PDF 157 kb)

Supplementary material 7 (PDF 221 kb)

Supplementary material 8 (PDF 163 kb)

Supplementary material 9 (PDF 152 kb)
