# Supplementary material for: Extended metAFLP approach in studies of tissue culture induced variation (TCIV) in triticale
Source: Mol Breed. 2014 May 7;34(3):845–54. doi: 10.1007/s11032-014-0079-2 (PMC4162973; doi:10.1007/s11032-014-0079-2)
Supplement: Supplementary file 2 — Supplementary material 2 (PDF 213 kb) [file 11032_2014_79_MOESM2_ESM.pdf]

Extended metAFLP approach in studies of the tissue culture induced variation (TCIV) in case of triticale

Molecular Breeding

Joanna Machczyńska[1], Renata Orłowska[1], Janusz Zimny[2], Piotr Tomasz Bednarek*[1]

[1]Department of Plant Physiology and Biochemistry

[2]Department of Plant Biotechnology and Cytogenetics

Plant Breeding and Acclimatization Institute - National Research Institute, 05-870 Błonie, Radzików, Poland

*Corresponding author: Piotr Tomasz Bednarek - p.bednarek@ihar.edu.pl

Online Resource 2 Oligonucleotides exploited for metAFLP in studies on triticale cv. Bogo.

**MetAFLP oligomer**	**Sequence 5’→ 3’**
Adapter1 Acc65I	CTCGTAGCATGCGTACA
Adapter2 Acc65I	GTACTGTACGCATGCTAC
Adapter1 KpnI	CTCGTAGCATGCGTACAGTAC
Adapter2 KpnI	TGTACGCATGCTAC
Adapter1 Mse	TACTCAGGACTCATC
Adapter2 Mse	GAGTCCTGAGTAGCAG
**Preselective primers**	
Acc65I/KpnI	GCATGCGTACAGTACC
MseI	GATGAGTCCTGAGTAAC
**Labeled ^{32}P selective oligonucleotides**	

CpG GCA	CATGCGTACAGTACCGCA
CpG GGC	CATGCGTACAGTACCGGC
CpG GAC	CATGCGTACAGTACCGAC
CpG ACG	CATGCGTACAGTACCACG
CpG TCG	CATGCGTACAGTACCTCG
CpXpG ATG	CATGCGTACAGTACCATG
CpXpG (A/T)GG	CATGCGTACAGTACC(A/T)GG
CpXpG AGG	CATGCGTACAGTACCAGG
CpXpG AGA	CATGCGTACAGTACCAGA
CpXpG AGC	CATGCGTACAGTACCAGC
CpXpG TGC	CATGCGTACAGTACCTGC
CpXpG TTG	CATGCGTACAGTACCTTG
CpXpX ATT	CATGCGTACAGTACCATT
CpXpX TAA	CATGCGTACAGTACCTAA
**Selective oligonucleotides**	
M CTC	GATGAGTCCTGAGTAACTC
M CTG	GATGAGTCCTGAGTAACTG
M CTA	GATGAGTCCTGAGTAACTA
M CTT	GATGAGTCCTGAGTAACTT
M CAC	GATGAGTCCTGAGTAACAC
M CAG	GATGAGTCCTGAGTAACAG
M CAT	GATGAGTCCTGAGTAACAT
M CAA	GATGAGTCCTGAGTAACAA
M CGC	GATGAGTCCTGAGTAACGC
M CGT	GATGAGTCCTGAGTAACGT

M CCG	GATGAGTCCTGAGTAACCG
M CCT	GATGAGTCCTGAGTAACCT
