# Supplementary material for: Extended metAFLP approach in studies of tissue culture induced variation (TCIV) in triticale
Source: Mol Breed. 2014 May 7;34(3):845–54. doi: 10.1007/s11032-014-0079-2 (PMC4162973; doi:10.1007/s11032-014-0079-2)
Supplement: Supplementary file 3 — Supplementary material 3 (PDF 187 kb) [file 11032_2014_79_MOESM3_ESM.pdf]

Extended metAFLP approach in studies of the tissue culture induced variation (TCIV) in case of triticale

Molecular Breeding

Joanna Machczyńska[1], Renata Orłowska[1], Janusz Zimny[2], Piotr Tomasz Bednarek*[1]

[1]Department of Plant Physiology and Biochemistry

[2]Department of Plant Biotechnology and Cytogenetics

Plant Breeding and Acclimatization Institute - National Research Institute, 05-870 Błonie, Radzików, Poland

*Corresponding author: Piotr Tomasz Bednarek - p.bednarek@ihar.edu.pl

Online Resource 3 Illustration of the different specificity of the *Acc65*I and *Kpn*I isoschizomers. Both endonucleases recognize the same DNA sequence (indicated in green and red), however, methylated site cannot be digested by *Acc65*I while can be hydrolyzed by *Kpn*I one. When the site is not methylated both enzymes may cut the DNA releasing different overlapping ends.

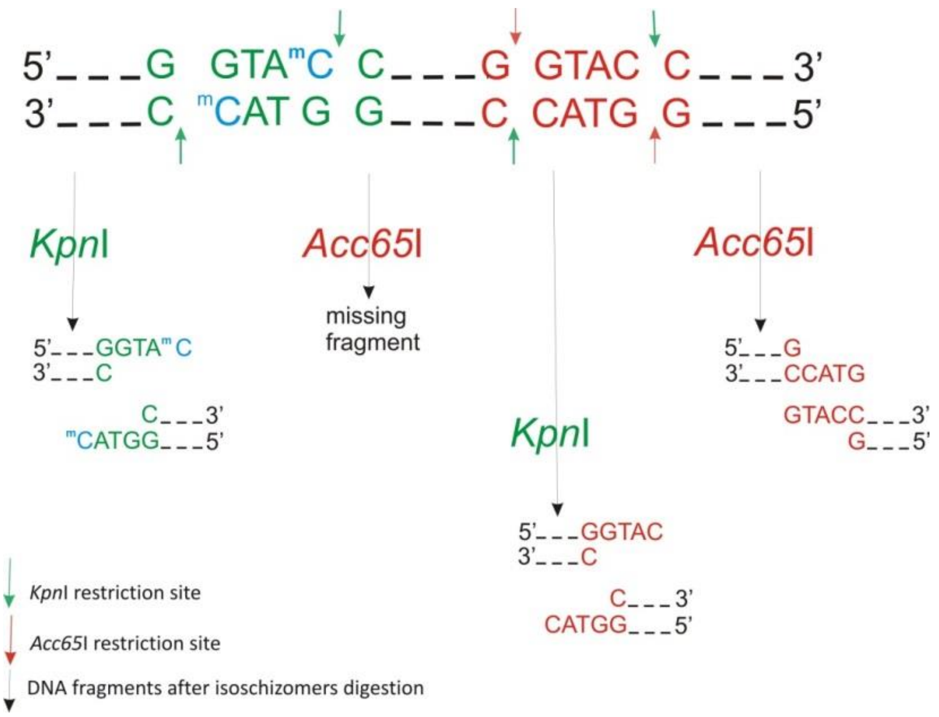

5'_ _ _G GTAᵐC C_ _ _G GTAC C_ _ _3'
3'_ _ _C ᵐCAT G G_ _ _C CATG G_ _ _5'
KpnI
Acc65I
Acc65I
missing fragment
5'_ _ _GGTAᵐC
3'_ _ _C
C_ _ _3'
ᵐCATGG_ _ _5'
KpnI
5'_ _ _GGTAC
3'_ _ _C
C_ _ _3'
CATGG_ _ _5'
5'_ _ _G
3'_ _ _CCATG
GTACC_ _ _3'
G_ _ _5'
KpnI restriction site
Acc65I restriction site
DNA fragments after isoschizomers digestion
