# Supplementary material for: Extended metAFLP approach in studies of tissue culture induced variation (TCIV) in triticale
Source: Mol Breed. 2014 May 7;34(3):845–54. doi: 10.1007/s11032-014-0079-2 (PMC4162973; doi:10.1007/s11032-014-0079-2)

Extended metAFLP approach in studies of the tissue culture induced variation (TCIV) in case of triticale

Molecular Breeding

Joanna Machczyńska[1], Renata Orłowska[1], Janusz Zimny[2], Piotr Tomasz Bednarek*[1]

[1]Department of Plant Physiology and Biochemistry

[2]Department of Plant Biotechnology and Cytogenetics

Plant Breeding and Acclimatization Institute - National Research Institute, 05-870 Błonie, Radzików, Poland

*Corresponding author: Piotr Tomasz Bednarek - p.bednarek@ihar.edu.pl

Online Resource 4 Explanation of the genetic background of the '1001' 4-digit code. Such a situation may take place only if the DNA of a donor plant has two consecutive restriction sites recognized by *Acc65*I and *Kpn*I endonucleases but the inner site is methylated (in red) and the outer one (in blue) is not. In case of the same fragment in the regenerant, the inner *Acc65*I-*Kpn*I (in grey) site is missing due to sequence change while the outer one is *de novo* methylated. Digestion of the donor and regenerant DNAs by the two AFLP platforms will result in '1001' and '0010' codes; however, the former reflects defined sequence change and *de novo* methylation that have to take place simultaneously.

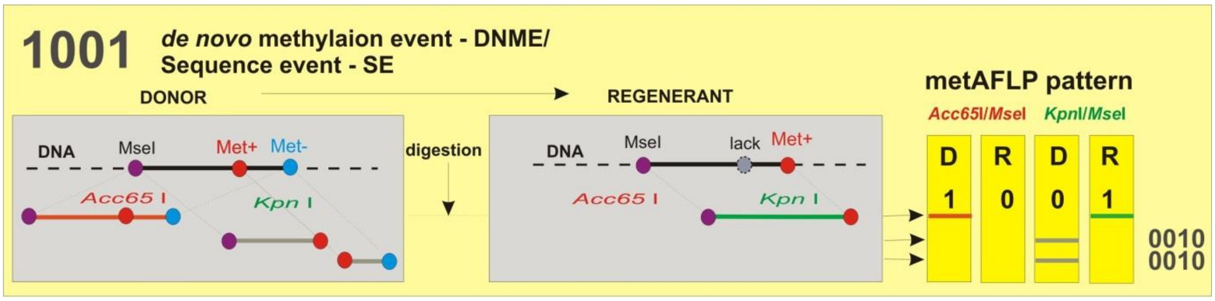

Supplement: Supplementary file 4 — Supplementary material 4 (PDF 204 kb) [file 11032_2014_79_MOESM4_ESM.pdf]
