# Supplementary material for: Extended metAFLP approach in studies of tissue culture induced variation (TCIV) in triticale
Source: Mol Breed. 2014 May 7;34(3):845–54. doi: 10.1007/s11032-014-0079-2 (PMC4162973; doi:10.1007/s11032-014-0079-2)

Extended metAFLP approach in studies of the tissue culture induced variation (TCIV) in case of triticale

Molecular Breeding

Joanna Machczyńska[1], Renata Orłowska[1], Janusz Zimny[2], Piotr Tomasz Bednarek*[1]

[1]Department of Plant Physiology and Biochemistry

[2]Department of Plant Biotechnology and Cytogenetics

Plant Breeding and Acclimatization Institute - National Research Institute, 05-870 Błonie, Radzików, Poland

*Corresponding author: Piotr Tomasz Bednarek - p.bednarek@ihar.edu.pl

Online Resource 5 Explanation of the genetic background of the '0110' 4-digit code. Such a situation may take place only if the DNA of a donor plant has a single restriction site recognized by *Acc65*I-*Kpn*I endonucleases, and it is methylated (in red). In case of the same fragment in the regenerant two consecutive restriction sites recognized by *Acc65*I and *Kpn*I endonucleases are present. The inner site is methylated (in red), and the outer (in blue) one is not. Digestion of the donor and regenerant DNAs by the two AFLP platforms will result in '0110' and '0001' codes; however, the former one reflect sequence change and demethylation taking place simultaneously.

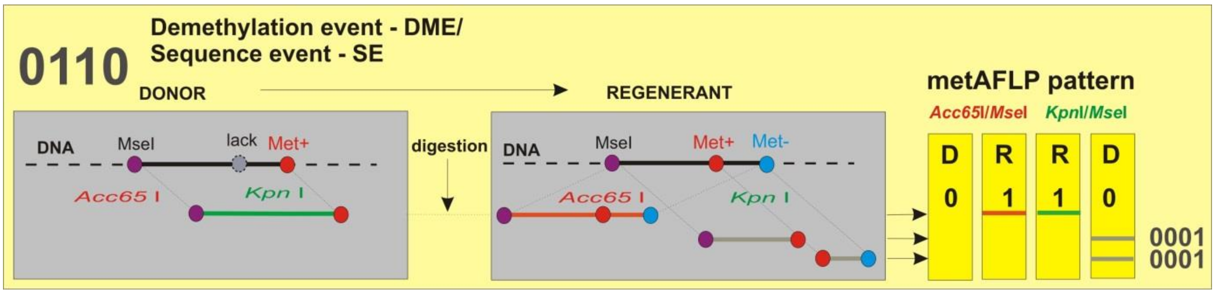

Supplement: Supplementary file 5 — Supplementary material 5 (PDF 131 kb) [file 11032_2014_79_MOESM5_ESM.pdf]
