# Supplementary material for: Extended metAFLP approach in studies of tissue culture induced variation (TCIV) in triticale
Source: Mol Breed. 2014 May 7;34(3):845–54. doi: 10.1007/s11032-014-0079-2 (PMC4162973; doi:10.1007/s11032-014-0079-2)
Supplement: Supplementary file 6 — Supplementary material 6 (PDF 157 kb) [file 11032_2014_79_MOESM6_ESM.pdf]

Extended metAFLP approach in studies of the tissue culture induced variation (TCIV) in case of triticale

Molecular Breeding

Joanna Machczyńska[1], Renata Orłowska[1], Janusz Zimny[2], Piotr Tomasz Bednarek*[1]

[1]Department of Plant Physiology and Biochemistry

[2]Department of Plant Biotechnology and Cytogenetics

Plant Breeding and Acclimatization Institute - National Research Institute, 05-870 Błonie, Radzików, Poland

*Corresponding author: Piotr Tomasz Bednarek - p.bednarek@ihar.edu.pl

Online Resource 6 Arrangement of selective primer combinations and the number of fragments amplified. TNAF – total number of amplified fragments; PFA – number of polymorphic fragments in the *Acc*65I/*Mse*I platform; PFK – number of polymorphic fragments in the *Kpn*I/*Mse*I platform; MFA – number of monomorphic fragments in the *Acc*65I/*Mse*I platform; MFK – number of monomorphic fragments in *Kpn*I/*Mse*I platform.

Selective primer combination	TNAF	PFA	PFK	MFA	MFK
**CpG GCA/M CGC**	28	11	6	17	22
**CpG GGC/M CTG**	32	21	22	11	10
**CpG GAC/M CAA**	42	25	21	17	21
**CpG ACG/M CAT**	40	12	7	28	33
**CpG TCG/M CGT**	47	34	29	13	18
**CpXpG ATG/M CTC**	43	15	15	28	28

**CpXpG (A/T) GG/M CCT**	48	16	11	32	37
**CpXpG AGG/M CAG**	33	15	18	18	15
**CpXpG AGA/M CAA**	51	29	26	22	25
**CpXpG AGC/M CTA**	37	18	13	19	24
**CpXpG TGC/M CCG**	43	28	24	15	19
**CpXpG TTG/M CTT**	47	18	22	29	25
**CpXpX ATT/M CAC**	49	33	34	16	15
**CpXpX TAA/M CGT**	44	35	30	9	14
**Total**	**584**	**310**	**278**	**274**	**306**
