# Supplementary material for: Extended metAFLP approach in studies of tissue culture induced variation (TCIV) in triticale
Source: Mol Breed. 2014 May 7;34(3):845–54. doi: 10.1007/s11032-014-0079-2 (PMC4162973; doi:10.1007/s11032-014-0079-2)
Supplement: Supplementary file 7 — Supplementary material 7 (PDF 221 kb) [file 11032_2014_79_MOESM7_ESM.pdf]

Extended metAFLP approach in studies of the tissue culture induced variation (TCIV) in case of triticale

Molecular Breeding

Joanna Machczyńska[1], Renata Orłowska[1], Janusz Zimny[2], Piotr Tomasz Bednarek*[1]

[1]Department of Plant Physiology and Biochemistry

[2]Department of Plant Biotechnology and Cytogenetics

Plant Breeding and Acclimatization Institute - National Research Institute, 05-870 Błonie, Radzików, Poland

*Corresponding author: Piotr Tomasz Bednarek - p.bednarek@ihar.edu.pl

Online Resource 7 Arrangement of events represented by individual 4-digit codes for regenerants derived via anther (RA), shed-microspore (RM), immature zygotic embryo cultures (RE) and for all regenerants taken together (Σ).

4-digit code	RA	RM	RE	∑
**0000**	1046	1225	1106	3377
**0001**	182	204	165	551
**0010**	266	344	316	926
**0011**	429	563	349	1341
**0100**	119	199	73	391
**0101**	228	262	294	784
**0110**	66	92	31	189
**0111**	539	561	656	1756

**1000**	192	220	238	650
**1001**	43	83	50	176
**1010**	151	238	167	556
**1011**	339	491	378	1208
**1100**	168	217	117	502
**1101**	272	290	297	859
**1110**	275	350	298	923
**1111**	10285	12181	10649	33115
