# Supplementary material for: Extended metAFLP approach in studies of tissue culture induced variation (TCIV) in triticale
Source: Mol Breed. 2014 May 7;34(3):845–54. doi: 10.1007/s11032-014-0079-2 (PMC4162973; doi:10.1007/s11032-014-0079-2)
Supplement: Supplementary file 8 — Supplementary material 8 (PDF 163 kb) [file 11032_2014_79_MOESM8_ESM.pdf]

Extended metAFLP approach in studies of the tissue culture induced variation (TCIV) in case of triticale

Molecular Breeding

Joanna Machczyńska[1], Renata Orłowska[1], Janusz Zimny[2], Piotr Tomasz Bednarek*[1]

[1]Department of Plant Physiology and Biochemistry

[2]Department of Plant Biotechnology and Cytogenetics

Plant Breeding and Acclimatization Institute - National Research Institute, 05-870 Błonie, Radzików, Poland

*Corresponding author: Piotr Tomasz Bednarek - p.bednarek@ihar.edu.pl

Online Resource 8 Arrangement of the *F* statistic values based on analysis of variance for variation types. Significance at $p<0.01$ and $\alpha=0.05$. RA, RM, RE – regenerants derived from anther cultures, shed microspore cultures and immature zygotic embryo cultures, respectively.

Model	RA	RM	RE	All regenerants
			***F* value**	
**SV - DMV - DNMV**	525.5	435.1	1141	1572
**CSV- CDMV - CDNMV**	554.9	443.6	1328	1647
