# Supplementary material for: Extended metAFLP approach in studies of tissue culture induced variation (TCIV) in triticale
Source: Mol Breed. 2014 May 7;34(3):845–54. doi: 10.1007/s11032-014-0079-2 (PMC4162973; doi:10.1007/s11032-014-0079-2)
Supplement: Supplementary file 9 — Supplementary material 9 (PDF 152 kb) [file 11032_2014_79_MOESM9_ESM.pdf]

Extended metAFLP approach in studies of the tissue culture induced variation (TCIV) in case of triticale

Molecular Breeding

Joanna Machczyńska[1], Renata Orłowska[1], Janusz Zimny[2], Piotr Tomasz Bednarek*[1]

[1]Department of Plant Physiology and Biochemistry

[2]Department of Plant Biotechnology and Cytogenetics

Plant Breeding and Acclimatization Institute - National Research Institute, 05-870 Błonie, Radzików, Poland

*Corresponding author: Piotr Tomasz Bednarek - p.bednarek@ihar.edu.pl

Online Resource 9 Variation grouping based on Tukey's test. The probability level for all statistical analyses was $p<0.01$ and $\alpha=0.05$. RA, RM, RE – regenerants derived from anther cultures, shed-microspores cultures and immature zygotic embryo cultures, respectively.

**Variation types**		**Tukey's grouping**			
		**RA**	**RM**	**RE**	**All regenerants**
**Uncorrected for complex variation**	**SV**	C	B	C	C
	**DMV**	B	A	B	B
	**DNMV**	A	A	A	A
		**RA**	**RM**	**RE**	**All regenerants**
**Corrected for complex variation**	**CSV**	C	B	C	C
	**CDMV**	B	A	B	B
	**CDNMV**	A	A	A	A
